# Efficacy and safety of ciprofol for general anesthesia in pediatric strabismus correction surgery: a prospective, randomized, double-blind, non-inferiority study

**DOI:** 10.3389/fmed.2026.1790750

**Published:** 2026-03-10

**Authors:** Wei Xu, Hongyi Xiao, Xinyuan Shi, Dandan Sun, Hongfa Xu, Chunfeng Zhao, Fanceng Ji

**Affiliations:** 1 School of Anesthesiology, Shandong Second Medical University, Weifang, China; 2 Department of Anesthesiology, Weifang Eye Hospital, Weifang, China; 3 Department of Anesthesiology, Weifang People's Hospital, Weifang, China

**Keywords:** children, ciprofol, general anesthesia, propofol, strabismus correction surgery

## Abstract

**Objective:**

To investigate the efficacy and safety of ciprofol for general anesthesia in pediatric strabismus correction surgery.

**Patients and methods:**

A total of 108 pediatric patients aged 4–12 years scheduled for strabismus correction surgery were randomly divided into two groups: the ciprofol group (Group C) and the propofol group (Group P). For anesthesia induction, Group C received an intravenous injection of ciprofol 0.5 mg/kg, alfentanil 20 μg/kg, and vecuronium bromide 0.05 mg/kg; Group P received an intravenous injection of propofol 2 mg/kg, alfentanil 20 μg/kg, and vecuronium bromide 0.05 mg/kg. After successful induction, a laryngeal mask airway was inserted and mechanical ventilation was initiated. For anesthesia maintenance, Group C was given a continuous intravenous infusion of ciprofol 1.25 mg/kg/h and alfentanil 40 μg/kg/h, while Group P received a continuous intravenous infusion of propofol 5 mg/kg/h and alfentanil 40 μg/kg/h. At the end of surgery, the infusion of anesthetic drugs was stopped, and the laryngeal mask airway was removed after the patients regained consciousness. The primary outcome measure was the anesthesia success rate. The secondary outcome measures included hemodynamic changes at different time points, induction time, emergence time, and the incidence of perioperative adverse events.

**Results:**

The anesthesia success rate was 100% in both groups. There were no statistically significant differences in hemodynamic changes at different time points, operation time, and emergence time between the two groups (*p* > 0.05). The incidence of adverse reactions in Group C was significantly lower than that in Group P (20.00% vs. 59.18%, *p* < 0.05).

**Conclusion:**

Ciprofol can be safely used for general anesthesia in pediatric strabismus correction surgery, with non-inferior anesthesia success rate compared with propofol and a lower incidence of adverse reactions.

**Clinical trial registration:**

https://www.chictr.org.cn, identifier ChiCTR2300078654.

## 1 Introduction

Pediatric strabismus is an ocular disorder characterized by misalignment of the visual axes of both eyes, caused by congenital developmental anomalies or extraocular muscle dysfunction, and some pediatric patients require surgical correction (1). Strabismus correction surgery features a limited surgical field of view and delicate manipulations, making satisfactory anesthetic efficacy crucial for the success of the procedure. As a commonly used clinical anesthetic, propofol is regarded as a relatively ideal option due to its rapid onset and fast recovery. However, propofol is associated with drawbacks such as injection pain and hemodynamic instability (2). Ciprofol is the first domestically developed intravenous anesthetic in China, which exhibits the characteristics of rapid onset, fast recovery, high potency and low incidence of injection pain (3, 4). Currently, ciprofol approved for use in China has been widely applied in digestive endoscopy, bronchoscopy, as well as general anesthesia induction and maintenance. Although existing studies on ciprofol in pediatric patients have provided preliminary references for clinical application, they are limited by flawed design, narrow clinical scenarios, and non-targeted dose selection, which are insufficient to support its standardized use across various pediatric surgeries. To date, most studies of ciprofol in pediatric anesthesia have focused on safety, dose exploration, and the prevention of specific adverse reactions, and were mainly conducted in short otolaryngological procedures (5). None of them systematically investigated anesthesia success rate as a primary endpoint, nor have they been applied in delicate ophthalmic surgeries requiring strict intraoperative immobility. Thus, the clinical application of ciprofol in pediatric anesthesia remains largely unexplored. Therefore, we hypothesized that ciprofol is non-inferior to propofol in terms of anesthesia success rate for pediatric strabismus correction surgery. We designed a prospective, randomized, double-blind controlled study to provide a reference for the clinical application of ciprofol in pediatric operations.

## 2 Methodology

The manuscript was written in accordance with the CONSORT statement guideline for a randomized controlled trial.

### 2.1 Ethical considerations

This study was conducted in strict accordance with the Declaration of Helsinki. The study protocol was approved by the Ethics Committee of Weifang Eye Hospital (Ethics Approval No. 2023-Institutional Ethics-04-01) and registered at the Chinese Clinical Trial Registry on December 14, 2023 (Registration No. ChiCTR2300078654). The study was carried out in the Department of Anesthesiology, Weifang Eye Hospital from January to July 2024, and written informed consent was obtained from the parents or legal guardians of all participants. No funding was received during the trial period.

### 2.2 Patient inclusion criteria

A total of 108 pediatric patients scheduled for elective strabismus correction surgery under general anesthesia were enrolled in this study. Inclusion criteria: Children aged 4–12 years with American Society of Anesthesiologists (ASA) physical status classification I–II. Exclusion criteria: A history of strabismus correction surgery; preoperative hypersensitivity to the study drugs; presence of significant medical history or incomplete data. Withdrawal criteria: Occurrence of serious adverse events or severe adverse reactions; withdrawal application submitted by the patients’ parents or legal guardians.

### 2.3 Randomization and blinding

This study was a prospective, randomized, double-blind, non-inferiority trial. Using a random number table method, researchers who only participated in the randomization process allocated patients to either the ciprofol group (Group C) or the propofol group (Group P) at a 1:1 ratio, with 54 cases in each group. Allocation concealment was achieved using sequentially numbered, opaque sealed envelopes. The study drugs were prepared by personnel not involved in data collection, based on the grouping information contained in the envelopes. Both ciprofol and propofol are white emulsions, and equal volumes of the two drugs were administered in this study. Patients, anesthesiologists, and researchers responsible for postoperative follow-up and data analysis were all blinded to the grouping assignments.

### 2.4 Methods of anesthesia

All patients underwent routine preoperative fasting and fluid deprivation. Upon entering the operating room, electrocardiogram (ECG), non-invasive blood pressure (NIBP), heart rate (HR), pulse oxygen saturation (SpO₂), and bispectral index (BIS) monitoring were performed for the patients. A unilateral intravenous access was established in the arm for Ringer’s solution infusion, and preoxygenation was conducted via a face mask with pure oxygen at a flow rate of 5 L/min.

Anesthesia induction in Group C, patients received an intravenous injection of ciprofol 0.5 mg/kg (administered within 1 min) plus alfentanil 20 μg/kg (administered within 30 s) plus vecuronium bromide 0.05 mg/kg. In Group P, patients received an intravenous injection of propofol 2.0 mg/kg (administered within 1 min) plus alfentanil 20 μg/kg (administered within 30 s) plus vecuronium bromide 0.05 mg/kg. If the patient’s eyelash reflex did not disappear within 2 min after induction, an additional dose of ciprofol 0.25 mg/kg or propofol 1 mg/kg was administered. If the patient still failed to fall asleep within 1 min after the first supplementary dose, a second supplementary dose was given. Induction failure was defined if the patient remained awake after two supplementary doses, and the anesthetic agent needed to be replaced. Artificial assisted ventilation was initiated once the eyelash reflex disappeared. Three minutes after administration, the degree of neuromuscular blockade reached the clinically effective level (1–2 twitches monitored by train-of-four (TOF) stimulation, meeting the airway requirements for laryngeal mask insertion). A laryngeal mask airway (LMA) was inserted, and controlled ventilation was maintained via an anesthesia machine. Anesthesia maintenance Group C was given a continuous intravenous infusion of ciprofol 1.25 mg/kg/h plus alfentanil 40 μg/kg/h. Group P was given a continuous intravenous infusion of propofol 5 mg/kg/h plus alfentanil 40 μg/kg/h. All surgeries were performed by the same surgeon. If intraoperative intraoperative movement occurred, an intravenous bolus of ciprofol 0.25 mg/kg (0.1 mL/kg) or propofol 1 mg/kg (0.1 mL/kg) was administered as a supplement. Maintenance failure was defined if more than two supplementary doses were required within 10 min. If blood pressure decreased by more than 30% from the baseline value, norepinephrine was administered; if bradycardia occurred, atropine was administered. In this study, a bispectral index (BIS) of 40–60 was used as the threshold for regulating anesthetic depth. Rescue measures to deepen anesthesia were planned if the BIS value exceeded this range. At the end of surgery, the infusion of anesthetic drugs was discontinued. The laryngeal mask airway was removed after the patient regained consciousness, and the patient was transferred to the post-anesthesia care unit (PACU).

### 2.5 Observation indicators

Outcome measures primary outcome measure: Anesthesia success rate. Secondary outcome measures: (1) Hemodynamic changes and bispectral index (BIS) values at time points from T0 to T6. (2) Anesthesia induction time, anesthesia emergence time, and incidence of perioperative adverse events, including intraoperative movement response during laryngeal mask insertion (defining non-reflexive, active intraoperative movements during laryngeal mask insertion, including head and neck twisting, limb flexion/extension, kicking, body lifting, and other movements), injection pain (referring to behaviors such as fist-clenching, leg-kicking, crying or verbal expression of pain exhibited by pediatric patients during intravenous drug administration), and incidence of nausea and vomiting within 24 h after surgery. Hemodynamic changes were observed and recorded at the following time points: before the initiation of anesthesia induction (T0), after the disappearance of eyelash reflex (T1), after laryngeal mask airway insertion (T2), at the start of surgery (T3), at 5 min after the start of surgery (T4), at the end of surgery (T5), and at the time of laryngeal mask airway removal (T6).

### 2.6 Sample size calculation

In this study, anesthesia success rate was set as the primary outcome measure, and sample size was calculated using a non-inferiority trial design. According to common practice in similar non-inferiority studies in the field of anesthesia, multiple clinical trials of ciprofol and propofol for anesthesia and sedation adopted −8% as the non-inferiority margin (2, 6). Meanwhile, combined with the commonly used range of −5 to −10% for non-inferiority margins in pediatric anesthesia studies (5, 7), the preset non-inferiority margin *δ* in this study was −8%. One-sided type I error *α* = 0.025, type II error *β* = 0.20 (power = 80%). Assuming that the anesthesia success rate was 98% in both the experimental group and the control group, with a 1:1 group ratio, the required sample size calculated by PASS 15.0 software was 98 patients. Considering an approximately 10% dropout rate, 54 patients were finally enrolled in each group, with a total of 108 patients.

### 2.7 Statistical analysis

Statistical analyses were performed using R software. The normality of continuous variables was assessed via the Shapiro–Wilk test. Normally distributed variables were expressed as mean ± standard deviation and compared using the Student’s *t*-test, whereas non-normally distributed variables were presented as median (interquartile range) and analyzed using the Mann–Whitney *U* test. Categorical variables were described as frequency (percentage) and compared using the Pearson’s chi-square test. A two-tailed *p*-value <0.05 was considered statistically significant.

## 3 Results

### 3.1 Patient condition

**Figure 1:**
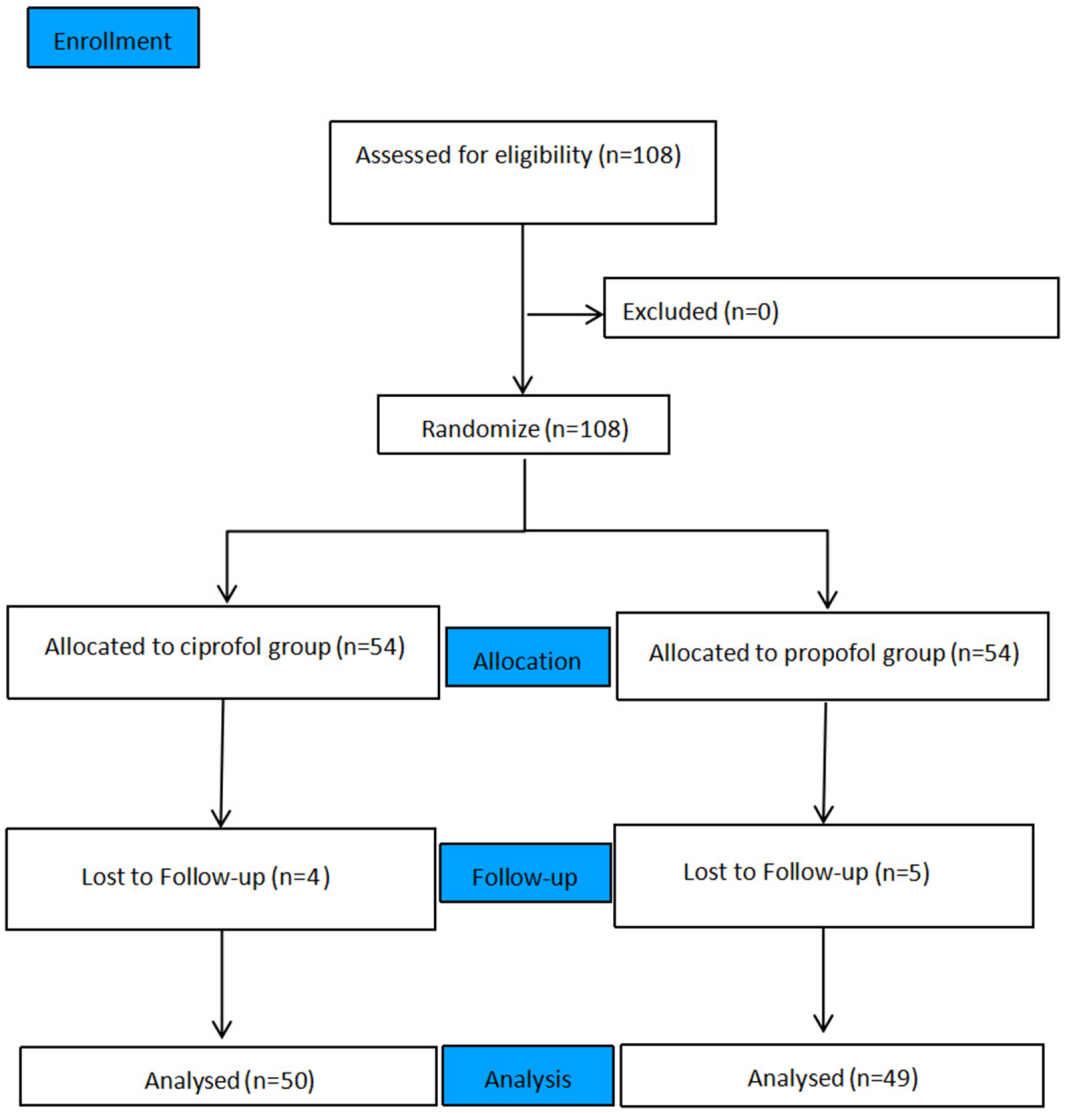
Flow chart of the experiment.

A total of 108 children were enrolled in this study. Among them, four patients in Group C and five patients in Group P were lost to follow-up. Ultimately, 99 children were included in the statistical analysis: 50 in Group C and 49 in Group P (see Figure 1).

### 3.2 Primary outcome

The anesthetic success rate was 100% in both groups (Table 1), the between-group difference was 0%. According to the criteria for non-inferiority testing, the lower limit of the 95% confidence interval for the between-group difference in anesthesia success rate was higher than the preset non-inferiority margin of −8%, confirming that ciprofol was non-inferior to propofol in terms of anesthesia success rate.

**Table 1:** Comparison of perioperative medication usage between groups.

Parameter	Group P	Group C	*p*-value
Propofol/Ciprofol induction dose (mg)	61.92 ± 24.29	16.66 ± 8.34	<0.001^*^
Alfentanil induction dose (mg)	0.61 ± 0.31	0.66 ± 0.21	0.350
Vecuronium induction dose (mg)	1.56 ± 0.78	1.66 ± 0.59	0.474
Propofol/Ciprofol maintenance dose (mg)	47.52 ± 22.63	13.81 ± 8.99	<0.001^*^
Alfentanil maintenance dose (mg)	0.4 (0.3, 0.5)	0.4 (0.3, 0.6)	0.308

Data are presented as mean ± standard deviation or median (interquartile range). * *p* < 0.05 indicates a statistically significant difference compared with Group P.

### 3.3 Secondary outcomes

There were no statistically significant differences between the two groups in terms of gender, age, body weight, BMI, or ASA physical status classification (Table 2). No statistically significant differences were observed in the duration of surgery or anesthesia emergence time between the two groups (Table 2). Additionally, there were no significant intergroup differences in hemodynamic parameters or bispectral index (BIS) values at time points T0 to T6 (Table 3). A significant difference was noted in the incidence of injection pain between the two groups (defined as adverse behavioral responses in children aged 4–12 years, including clenching fists, kicking legs, crying, or verbal reports of pain during injection): 16.00% in Group C versus 51.02% in Group P (*p* < 0.05). The incidence of intraoperative movement during laryngeal mask airway insertion was 4.00% in Group C (resolved within 30 s after additional administration of 5 mg ciprofol) versus 14.29% in Group P. The incidence of postoperative nausea and vomiting was 8.00% in Group C versus 4.08% in Group P. Compared with the propofol group, the ciprofol group had a significantly lower incidence of total adverse reactions: 20.00% versus 59.18% (*p* < 0.001) (Table 4).

**Table 2:** Comparison of general characteristics between groups.

Parameter	Group P	Group C	*p*-value
Number of cases (*n*)	49	50	—
Age (years, $\bar{x}\pm s$ )	8.06 ± 1.93	8.14 ± 2.22	0.849
Sex (male)	23 (46.9%)	24 (48.0%)	0.916
Weight (kg, $\bar{x}\pm s$ )	30.96 ± 12.74	33.52 ± 11.96	0.305
Height (cm, $\bar{x}\pm s$ )	133.08 ± 17.94	134.32 ± 21.05	0.752
BMI	17.05 ± 4.00	18.01 ± 3.32	0.197
Anesthesia duration (min)	20.49 ± 5.78	21.42 ± 6.97	0.471
Anesthesia emergence time (min)	10.00 ± 2.16	10.42 ± 3.31	0.455

Data are presented as mean ± standard deviation or *n* (percentage). BMI, body mass index.

**Table 3:** Comparison of MAP, HR, and BIS at various time points between groups.

Variable	Group	T0	T1	T2	T3	T4	T5	T6
MAP (mmHg)	Group P	78.92 ± 9.70	78.06 ± 9.74	67.86 ± 10.29	66.06 ± 9.59	66.47 ± 9.13	76.82 ± 10.85	87.27 ± 12.75
	Group C	80.28 ± 11.41	75.68 ± 11.72	67.54 ± 9.20	67.24 ± 8.89	67.30 ± 11.00	75.02 ± 9.75	84.38 ± 12.25
*p*-value		0.262	0.137	0.436	0.264	0.342	0.389	0.164
HR (bpm)	Group P	92.63 ± 11.68	83.41 ± 7.18	83.53 ± 9.38	84.69 ± 8.69	83.41 ± 11.73	87.27 ± 12.75	90.65 ± 13.02
	Group C	93.18 ± 14.61	82.76 ± 11.92	82.74 ± 12.94	82.22 ± 12.84	81.46 ± 9.83	84.38 ± 12.25	88.16 ± 13.21
*p*-value		0.427	0.538	0.59	0.163	0.397	0.407	0.883
BIS	Group P	93.41 ± 2.00	53.24 ± 10.38	60.94 ± 3.05	58.98 ± 4.45	56.98 ± 5.53	73.86 ± 6.75	82.08 ± 8.33
	Group C	93.10 ± 1.84	52.24 ± 4.89	60.60 ± 3.18	57.74 ± 4.33	56.26 ± 3.88	72.56 ± 8.62	81.88 ± 4.89
*p*-value		0.837	0.744	0.729	0.265	0.372	0.254	0.347

Data are presented as mean ± standard deviation. bpm, beats per minute.

**Table 4:** Comparison of perioperative adverse events between groups.

Parameter	Group P	Group C	*p*-value
Injection pain	25 (51.02%)	8 (16.00%)	<0.001^*^
Movement during LMA Insertion	7 (14.29%)	2 (4.00%)	0.087
PONV	2 (4.08%)	4 (8.00%)	0.494
Adverse reaction incidence rate	29 (59.18%)	10 (20.00%)	<0.001^*^

Data are presented as *n* (percentage). * *p* < 0.05 indicates a statistically significant difference compared with Group P. LMA, laryngeal mask airway; PONV, postoperative nausea and vomiting.

## 4 Discussion

In this study, anesthesia success rate was set as the primary outcome measure. As a fundamental and core clinical indicator for evaluating the efficacy of anesthetic agents, it directly reflects whether the drug meets the basic anesthetic requirements of surgery. Pediatric strabismus correction is a delicate elective ophthalmic procedure with a narrow surgical field and manipulations adjacent to the eyeball. Stable anesthesia induction and maintenance are critical for the successful completion of surgery, which provides an important rationale for selecting anesthesia success rate as the primary outcome in this study. Currently, clinical studies of ciprofol are mostly concentrated in the field of adult anesthesia, whereas relevant studies on its application in pediatric general anesthesia remain scarce. Sufficient evidence-based medical support is still lacking for its efficacy and safety in the pediatric population. However, multiple adult studies have confirmed the favorable performance of ciprofol in anesthesia success rate. For example, Zhang et al. (2) reported that both ciprofol and propofol achieved a 100% anesthesia success rate in adult gynecological day surgery, demonstrating that ciprofol was non-inferior to propofol. In addition, the multicenter randomized controlled study by Luo et al. (8) showed that ciprofol could achieve an ideal anesthesia success rate for sedation during adult fiberoptic bronchoscopy. Based on the above evidence, the present study hypothesized that ciprofol is non-inferior to propofol in anesthesia success rate during general anesthesia for pediatric strabismus correction surgery, and was designed as a prospective, randomized, double-blind, non-inferiority trial.

As a short-acting intravenous anesthetic structurally modified from propofol, ciprofol exhibits approximately 3-fold higher affinity for GABAA receptors and 4–5-fold greater potency than propofol (6). This key pharmacological profile provides an important basis for clinical dosage setting and efficacy comparison, and enables stable anesthetic effects at low doses. The results of the present study showed that the anesthesia success rate was 100% in both the ciprofol group and the propofol group, suggesting that ciprofol can stably modulate γ-aminobutyric acid receptors in the pediatric central nervous system (7) and achieve smooth anesthesia induction and intraoperative sedation by inhibiting neuronal excitability. This finding is highly consistent with the results reported by Zhao et al. (9) in pediatric adenotonsillectomy, further verifying the stable pharmacodynamic characteristics of ciprofol in pediatric general anesthesia. Meanwhile, the selection of anesthetic agents in pediatric practice requires minimizing the risk of adverse reactions on the premise of ensuring adequate anesthesia. In this study, the total incidence of adverse events in the ciprofol group was significantly lower than that in the propofol group (20.00% vs. 59.18%, *p* < 0.001). This advantage was mainly attributed to the pharmacological and formulation characteristics of ciprofol: First, the concentration of free drug in the aqueous phase of its lipid emulsion is lower (10), which greatly reduces direct stimulation to vascular endothelium and constitutes the main reason for the markedly lower incidence of injection pain. These results are consistent with those reported by Luo et al. (8) and Chen et al. (11). Second, its high potency allows satisfactory anesthesia to be achieved at low doses, reducing overall systemic stimulation to the central nervous and cardiovascular systems. Furthermore, there were no significant differences in mean arterial pressure (MAP) or heart rate (HR) between the two groups at any time point, confirming that ciprofol has mild effects on the pediatric cardiovascular system at equipotent anesthetic doses, with hemodynamic safety comparable to that of propofol.

The results of the present study are highly consistent with those of previous pediatric and adult studies on ciprofol, further confirming its universal advantages in clinical anesthesia. In the pediatric field, Zhao et al. (9) conducted a study in children aged 3–10 years with ASA class I–II undergoing adenotonsillectomy, using the same inclusion criteria and potency-matched dosage regimen as the present study. Their results showed a 100% success rate of anesthesia induction with ciprofol, a total incidence of adverse events of 22.3%, and an incidence of injection pain of 15.6%, which were highly close to the corresponding indicators in the ciprofol group of the present study (100, 20.00, 16.00%). These findings confirm that ciprofol can achieve anesthetic effects equivalent to propofol at low doses in different types of pediatric surgery, with the advantage of fewer adverse reactions. In the adult field, a systematic review by Marik (3) confirmed that ciprofol has a similar onset time to propofol, with a significantly lower incidence of adverse events such as hemodynamic fluctuation and injection pain. A multicenter study by Lu et al. (4) showed no difference in anesthesia induction success rate between ciprofol and propofol, with an injection pain incidence of only 5.2%. Man et al. (12) also demonstrated a 100% anesthesia success rate in adult gynecological day surgery, with significantly lower incidences of adverse events and postoperative nausea and vomiting (PONV). All the above studies confirmed that ciprofol is non-inferior to propofol in anesthetic efficacy at potency-matched doses, with fewer adverse reactions. The dosage difference between the present study and adult studies further verifies the high potency characteristic of ciprofol: adults require higher doses due to factors such as metabolic capacity and body weight, whereas children can achieve stable anesthetic effects at lower doses, which conforms to the unique pharmacokinetic characteristics of the pediatric population.

The slight differences between the present study and previous studies were not attributed to variations in the pharmacological effects of ciprofol, but rather to the specificity of surgical types and anesthetic requirements. Compared with pediatric adenotonsillectomy in Zhao et al. (9), strabismus correction surgery in this study demands deeper anesthesia and stricter intraoperative immobility. Even minor intraoperative movement may interfere with surgical procedures or even cause ocular injury. Therefore, the maintenance dose of ciprofol was slightly adjusted to 1.25 mg/kg/h in this study, which was slightly higher than the 1.0–1.2 mg/kg/h used in the previous study, to ensure adequate intraoperative sedation depth. This adjustment also suggests that the pediatric dosage of ciprofol should be individualized according to the delicacy of the surgery and specific anesthetic requirements.

Regarding postoperative nausea and vomiting (PONV), although Sahinovic et al. (13) and Xiao et al. (14) demonstrated that subhypnotic doses of propofol exert significant antiemetic effects, and ciprofol has also been reported to possess potential antiemetic properties (8), no significant difference in PONV incidence was observed between the two groups in the present study (8.00% vs. 4.08%), and the antiemetic advantage of ciprofol was not detected. This result differs from the study of Man et al. (12) in adult gynecological day surgery. The main reason lies in the different baseline risks of PONV related to surgical sites: adult gynecological surgery involves pelvic traction and stimulation, which are high-risk factors for PONV with a relatively high baseline incidence. In contrast, pediatric strabismus correction surgery is far from the digestive tract without abdominal or pelvic traction stimuli, leading to an overall low baseline PONV incidence. Thus, the potential antiemetic property of ciprofol was difficult to demonstrate. This finding is also consistent with the conclusion proposed by Shi et al. (15) that the antiemetic effect of low-dose ciprofol is related to surgical type.

This study has certain limitations. First, it adopted a single-center study design with a relatively limited sample size, which may lead to selection bias and thus fail to fully reflect clinical diversity. Second, the observation period was relatively short, and no follow-up was conducted to assess the long-term cognitive function and growth indicators of the children after surgery. Although existing studies have indicated that a single session of short-duration anesthesia exerts no significant impact on children’s brain development, targeted data supporting the long-term safety of ciprofol in this aspect are still lacking.

## 5 Conclusion

In conclusion, ciprofol can be safely used for general anesthesia in pediatric strabismus correction surgery. It is non-inferior to propofol in terms of anesthetic success rate and is associated with a lower incidence of adverse reactions.

## Data Availability

The original contributions presented in the study are included in the article/supplementary material, further inquiries can be directed to the corresponding authors.
